# Parkinsonism caused by Intracranial ependymoma: A rare case report and literature review

**DOI:** 10.1002/agm2.12093

**Published:** 2019-12-27

**Authors:** Yue Lou, Yanwen Wang, Liying Zhuang, Miao Cai, Xiaoli Liu

**Affiliations:** ^1^ Department of Neurology Zhejiang Hospital Hangzhou China

**Keywords:** ependymoma, parkinsonism, surgical resection

## Abstract

**Background:**

Ependymomas, especially intracranial ependymomas, are rare neoplasms of the CNS. The clinical courses of patients with intracranial ependymomas can be quite variable. At present, data on parkinsonism caused by ependymomas are scarce.

**Case presentation:**

A 13‐year‐old girl presented with parkinsonism symptoms of clumsiness in her left leg and hand. Her mother was diagnosed with Parkinson's disease at age 30, nine years previously. Magnetic resonance imaging showed a lesion in the temporal lobe with long‐T1 signal, mixed‐T2 signal. The patient was taken in for a right tumorectomy and was diagnosed as having an ependymoma postoperatively. The patient's symptoms fully resolved in the postoperative phase.

**Conclusion:**

The case describes the mechanism of intracranial ependymoma involving parkinsonism symptoms. Our findings suggest that in some patients presenting with atypical PD symptoms the underlying cause should not be overlooked; MRI examination is necessary.

**Abbreviations**
CNS: central nervous system
MRI: magnetic resonance imaging
PD: Parkinson's disease

## 1 INTRODUCTION

Parkinsonism is characterized by motor features such as bradykinesia, resting tremor, rigidity, postural instability and many non‐motor symptoms. Several etiologies such as heredity, head injury, infection, neurotoxin, and environmental factors have been proposed as playing a role in the causes of parkinsonism.^1^ The occurrence of Parkinson's syndrome is caused by dysfunction of the basal ganglia. The differentiation of idiopathic Parkinson's disease, hereditary Parkinson's disease and parkinsonism may be important for us to choose the right treatment. In this paper, we report a child with subacute extrapyramidal symptoms, whose mother was diagnosed with Parkinson's disease nine years before, resembling clinically hereditary Parkinson's disease. The patient was finally diagnosed as ependymoma by pathological examination, which was located in the right temporal lobe. To the best of our knowledge, this article is the first in the literature to document Parkinson‐like symptoms in temporal ependymoma.

## 2 CASE PRESENTATION

A 13‐year‐old girl was admitted to our neurology department because of inflexibility in her left leg and left hand when she was in physical education. The patient had noticed three months previously that her left leg got easily caught up in the rope when she was skipping because of its clumsiness A similar symptom appeared in her left hand one month ago: it took a lot of effort and time for her to do up buttons.

The patient was a middle school student and had performed very well in sports three months earlier. The patient's mother had a normal pregnancy. The girl was born at 39 weeks of gestation with a birthweight of 3100 g; her Apgar score was normal.

The patient has a family history: her mother was diagnosed with Parkinson's disease at the age of 30, nine years ago, with initial symptoms of bradykinesia and rigidity in her left arm, which then progressively affected the left leg, right arm and right leg. Her mother's symptoms improved significantly after taking L‐dopa 1 hour.

The patient's neurological examination showed mild bradykinesia and rigidity in her left arm and leg, her muscle strength is normal and Babinski sign is negative. Further physical examination revealed nothing abnormal: the blood pressure was 106/66 mm Hg, and her serum biochemistry, whole blood count, thyroid function were all normal. The patient had a related family history, suggesting that it might be hereditary PD. We applied genetic analysis to the proband (the patient) using targeted multiplex ligation‐dependent probe amplification (*MLPA*)+ next‐generation sequencing (*NGS*) to cover candidate genes known to cause familial forms of PD. We detected genes of interest including Recombinant Parkinson Disease Protein 2 (PARK2), Leucine‐rich repeat kinase 2 (LRRK2), ATPase cation transporting 13A2(ATP13A2), Alpha‐Synuclein (SNCA), ubiquitin C‐terminal hydrolase L1 (UCHL1), PTEN induced kinase 1 (PINK1), Parkinsonism associated deglycase (PARK7), ATPase copper transporting beta (ATP7B), GTP cyclohydrolase 1 (GCH1), GRB10 interacting GYF protein 2 (GIGYF2), HtrA serine peptidase 2 (HTRA2), phospholipase A2 group VI (PLA2G6), F‐box only protein 7 (FBXO7), vacuolar protein sorting 35 homolog gene (VPS35), vacuolar protein sorting 13 homolog C (VPS13C), vacuolar protein sorting 13 homolog A (VPS13A), eukaryotic translation initiation factor 4 gamma 1 (EIF4G1), DnaJ heat shock protein family (Hsp40) member C6 (DNAJC6), DnaJ heat shock protein family (Hsp40) member C13 (DNAJC13), synaptojanin 1 (SYNJ1), parkin coregulated (PACRG), interleukin 1 beta (IL1B), ataxin 2 (ATXN2), ataxin 3 (ATXN3), ATPase H+ transporting accessory protein 2 (ATP6AP2), RAB39B, Microtubule‐associated protein Tau (MAPT), TATA‐binding protein (TBP), prodynorphin (PDYN), transglutaminase 6 (TGM6), nuclear receptor subfamily 4 group A member 2 (NR4A2), glutamate dehydrogenase 2 (GLUD2), glucosylceramidase beta (GBA), pantothenate kinase 2 (PANK2), phospholipase A2 group VI (PLA2G6), ferritin light chain (FTL), WD repeat domain 45 (WDR45), Coenzyme A synthase (COASY), presenilin 1 (PSEN1), coenzyme Q2(COQ2), syntaxin 1B (STX1B), dynactin subunit 1 (DCTN1), DDRGK domain‐containing protein 1 (DDRGK1), TAR DNA‐binding protein 43 (TARDBP), ceruloplasmin (CP). The same Parkinsonism‐related gene panel was applied to her mother. However, no mutation gene was found in the patient and her mother.

In addition, the patient had normal liver function, no corneal KF ring, and no ATP7B gene mutation; therefore, we did not consider this patient as hepatolenticular degeneration (Wilson disease, WD). Moreover, the patient was not using dopamine receptor blockers, so drug‐induced Parkinson's syndrome was not considered. The patient also did not have fever, personality changes, cognitive impairment, joint pain or other immune‐related symptoms, autoimmune Parkinson's syndrome was not considered. She has not recently been infected or vaccinated, so infection‐related Parkinson's syndrome was not considered. Moreover, she denied exposure to toxic substances, so poisoning‐related Parkinson's syndrome was not considered.

**Figure 1:**
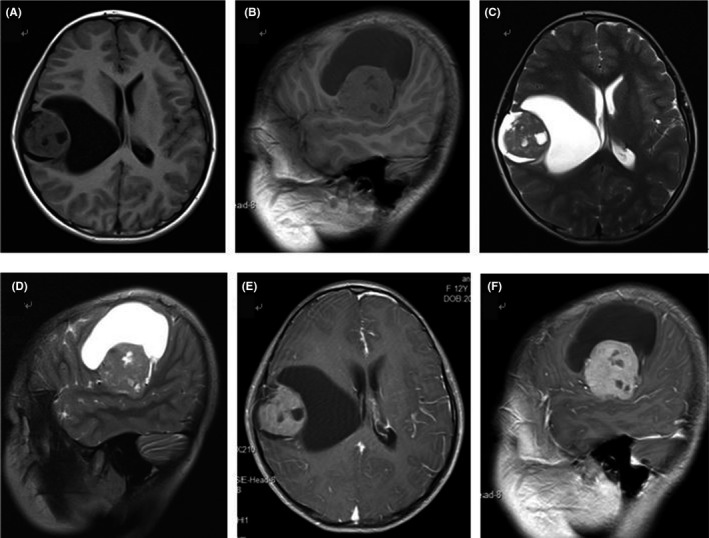
MRI showed a lesion 7.3 × 6.2 × 6.8 cm in size in the temporal lobe with mixed long‐T1 signal (a, b), mixed long‐T2 signal (c, d), and heterogeneous enhancement in the solid part (e, f)

Then, magnetic resonance imaging (MRI) was taken for the patient. MRI showed that there was a lesion about 7.3 × 6.2 × 6.8 cm in size in the temporal lobe with long mixed‐T1 signal, long mixed‐T2 signal, and there was cystic degeneration in periphery regions of the lesions. There was heterogeneous contrast enhancement in the solid part on gadolinium‐enhanced T1‐weighted images (Figure 1).

**Figure 2:**
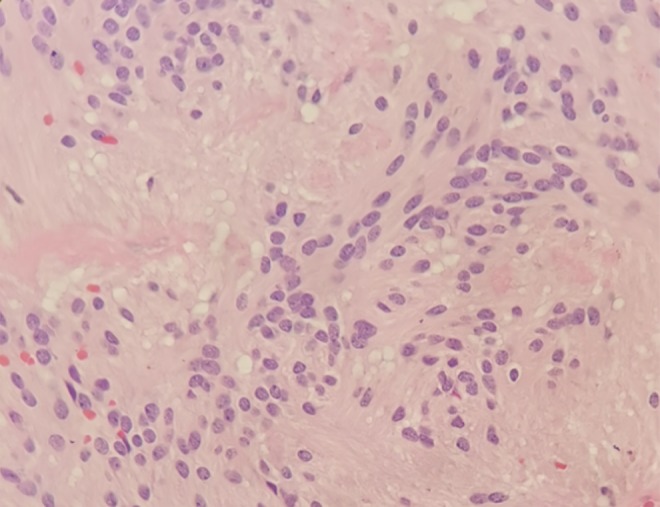
Histopathologic characterization of the ependymoma. the tumor cells forming perivascular pseudorosettes. (H & E, 400×)

The mass was completely removed via right tumorectomy, then microscopic examination revealed that the resected tumor cells stuck out of elongated cytoplasmic protuberances attached to the dilated radial arrangement around thin‐walled blood vessels to form perivascular pseudorosettes (Figure 2). The space around the vessels without cells was wide. Some tumor cells were large, cytoplasm was eosinophilic, and nuclear fission was rare. The pathological diagnosis revealed a typical ependymoma (WHO II). Immunohistochemical staining demonstrated that the tumors expressed glial fibrillary acid protein (GFAP), S‐100 protein, vimentin and epithelial membrane antigen (EMA) and p53 protein, which were weakly positive immunoreactivity for GFAP, S‐100, positive immunoreactivity for EMA and P53. The Ki‐67 was 10%‐30%.

**Figure 3:**
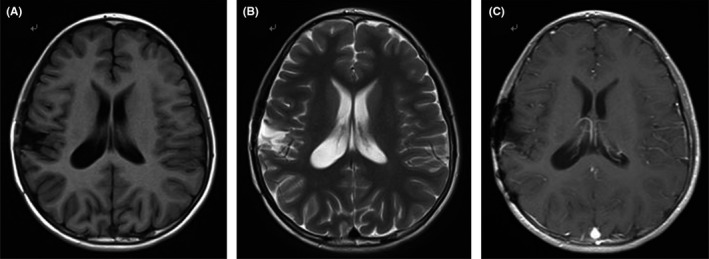
MRI after surgery showed partial cerebrospinal fluid retention in the right parietal lobe, T1‐weighted image (a), T2‐weighted image (b), enhanced T1WI (c)

The patient recovered well after the operations and her left leg and hand were almost as flexible as before. MRI after surgery showed that 33a cystic lesion with cerebrospinal fluid signal was left, and the cystic lesion was decreased two months later (Figure 3). After surgery, she received radiation therapy and there was no recurrence in the follow‐up study.

## 3 DISCUSSION AND CONCLUSION

Ependymomas are central nervous system (CNS) tumors that usually arise from the cell lining of the ventricle and the central canal of the spinal cord or the white matter ependymal cells in the brain, accounting for 3%‐5% of intracranial glial neoplasms.^2^ Ependymomas can arise throughout all compartments of the central nervous system, usually involving the three major anatomic compartments (supratentorial brain, posterior fossa, and spinal cord) with prevalence for intracranial and spinal location in children and adults, respectively, which occur at two major peaks in life around 0‐4 and 55‐59 years of age, respectively.^3^ Ependymomas mostly occur in the fourth ventricle, and may also occur in the lateral ventricle, brain parenchyma, spinal cord or cauda equine. In adults, the majority of ependymomas are located in the spine (SP, 46%),^4^ while pediatric ependymomas are almost entirely located intracranially (90%).^5^ According to https://www.ncbi.nlm.nih.gov/pubmed/?term=Cage%2520TA%255bAuthor%255d%26cauthor=true%26cauthor_uxml:id=23540528 research, out of 182 pediatric patients, 69% had supratentorial ependymomas and 31% presented with infratentorial lesions.^6^ The infratentorial extraventricular ependymomas are more often located in the cerebellar hemisphere, while the supratentorial ependymomas mainly occur in the brain parenchyma.^3^

At present, data on parkinsonism caused by ependymomas are scarce. We know Kalff reported a case of Parkinson's syndrome caused by ependymoma of the cauda equine,^7^ We are the first reported Parkinson's syndrome caused by ependymoma of the brain parenchyma.

The first symptom of patients with ependymomas is the intracranial hypertension for treatment. In this article, we report a case of Parkinsonian syndrome caused by temporal ependymoma. The patient was admitted to our hospital because of clumsiness in her left leg and left hand. Her neurological examination revealed some signs of PD. When we questioned the patient's medical history in detail, we found that the patient suffered from acute onset of left leg bradykinesia, which was an atypical onset form of PD. Moreover, her leg bradykinesia was worse within three months and similar symptom appeared in her left hand quickly, also atypical for PD. Sudden onset, rapid progression, and normal genetic analysis, together with the imaging findings and histopathologic characterization, support the diagnosis of Parkinsonian syndrome caused by ependymoma in our case. The cause of Parkinsonian syndrome in our case may be due to right temporal ependymoma disrupting the cortico‐basal ganglia‐cortical loop, then induced basal ganglia dysfunction, not being the result of a loss of nigral dopaminergic neurons.^8^

Pathologically, ependymomas are classified according to the World Health Organization (WHO) grading system. The WHO classification system separates ependymomas into grades I, II and III. Grade I ependymoma mainly occurs in adults and is associated with good clinical outcomes. Grade I tumors include myxopapillary ependymoma, which usually occurs in the spine, and subependymoma that usually occur in the brain.^9^ Grade II ependymoma shows pathologically perivascular pseudorosettes and characteristic true ependymal rosettes. Anaplastic ependymoma (grade III) is also called malignant ependymoma, which is characterized by hypercellularity, abundant mitotic activity, pseudoatrophic necrosis and microvascular proliferation.^10^ The diagnosis of ependymoma is usually made without difficulty, but is occasionally challenging when there is a background of Parkinson's Disease (PD), because of the tumor’s unusual features that include clinical manifestation and microscopically infiltrative growth and intermixed.^11^

Surgery plays a major role in local tumor control. In addition to surgery, postoperative radiotherapy at a dose of 54‐59.4 Gy is considered to be the standard treatment for reducing the risk of local recurrence in patients with non‐disseminated ependymoma.^9^, ^12^

Thus, this case indicates that detailed medical history and imaging information were important for the accurate diagnosis of PD, especially atypical PD, which could help us choose appropriate treatment strategies for patient. Our patient's mother had similar symptoms and had been diagnosed as having Parkinson's disease for many years; then the patient presented with rapid progress of Parkinson‐like symptoms, but the symptoms were atypical and genetic analysis was normal. Therefore, even if clinical symptoms, neurological examination and family history suggest hereditary Parkinson's disease, imaging examination is necessary and the underlying cause should not be overlooked.

## CONFLICTS OF INTEREST

Nothing to disclose.

## AUTHOR CONTRIBUTIONS

*Manuscript writing and interpretation of the data*: YL. *Acquisition and interpretation of the data*: YW and LZ. *Critical revision of the manuscript for intellectual content*: MC and XL. All authors read and approved the final manuscript.

## CONSENT TO PUBLISH

Written informed consent was obtained from the patient and her mother for publication of this case report and any accompanying images.

## Data Availability

All data generated during this study are available from the corresponding author upon reasonable request.
